# Not quite a cure yet: unlocking the unfulfilled promise of live biotherapeutics for disease treatment

**DOI:** 10.3389/fphar.2025.1695976

**Published:** 2025-11-05

**Authors:** Andrea Verdugo-Meza, Han M. Chiang, Emeran A. Mayer, Deanna L. Gibson

**Affiliations:** ^1^ Department of Biology, Faculty of Science, University of British Columbia, Okanagan Campus, Kelowna, BC, Canada; ^2^ Vatche & Tamar Manoukian Division of Digestive Diseases, UCLA and Goodman Luskin Microbiome Center UCLA, Los Angeles, CA, United States; ^3^ Melius MicroBiomics Inc., Kelowna, BC, Canada; ^4^ Department of Medicine, Faculty of Medicine, University of British Columbia, Vancouver, BC, Canada

**Keywords:** live biotherapeutic product, probiotic, inflammation, *Escherichia coli* nissle 1917 (EcN), live microbial therapeutics

## Abstract

Few live microbial therapeutics have demonstrated conclusive or reproducible clinical efficacy. Cochrane meta-analyses of probiotic interventions across multiple clinical trials, analyzed using a rank-biserial correlation test, revealed that intestinal inflammation is negatively correlated with clinical responsiveness. This is exemplified by *Escherichia coli* Nissle 1917, which shows efficacy in maintaining remission comparable to frontline therapies, yet fails to demonstrate clear benefit in the treatment of active ulcerative colitis, an inflammatory bowel disease defined by chronic intestinal inflammation. Beyond inflammation as a key barrier to efficacy, inadequate shelf life and delivery strategies further compromise microbial viability and functional persistence in the gastrointestinal tract. Here, we highlight both the challenges and emerging opportunities in the field of live microbial therapeutics, emphasizing the urgent need to integrate scientific, clinical, and industrial efforts to achieve durable and clinically meaningful outcomes.

## Overview

Probiotics and live biotherapeutic products (LBPs) differ fundamentally in their definitions, intended purposes, and regulatory classifications. While probiotics are dietary supplements or food additives aimed at promoting general or gut health in otherwise healthy populations, LBPs are developed and regulated as pharmaceutical drugs for the prevention or treatment of specific diseases under the authority of the U.S. Food and Drug Administration (FDA) (Heavey et al., 2022). Consequently, LBPs are restricted to microbial strains with demonstrated therapeutic efficacy in defined disease contexts, criteria that most conventional probiotics do not meet. Despite the FDA’s efforts in 2010 to delineate LBPs through a more rigorous regulatory pathway, many over-the-counter probiotics continue to make health claims targeting diseased populations, and several have been evaluated in clinical trials. There are only two approved LBPs: both are for *Clostridia difficile* infection (Bland et al., 2025 #734). Here we present our perspective after reviewing clinical trial and meta-analysis data for both probiotics and LBPs. This led to our conclusion that microbial therapeutics have shown limited efficacy in treating chronic intestinal inflammation. Given the same beneficial bacteria could be either a probiotic or a LBP, depending on the regulatory path taken, we extend our findings of probiotics tested in clinical trials to postulate why microbial therapeutics have yet to fulfill their promise and outline strategies for advancing the field to realize their full clinical potential.

## Intestinal inflammation undermines the promise of live biotherapeutic products

**FIGURE 1:**
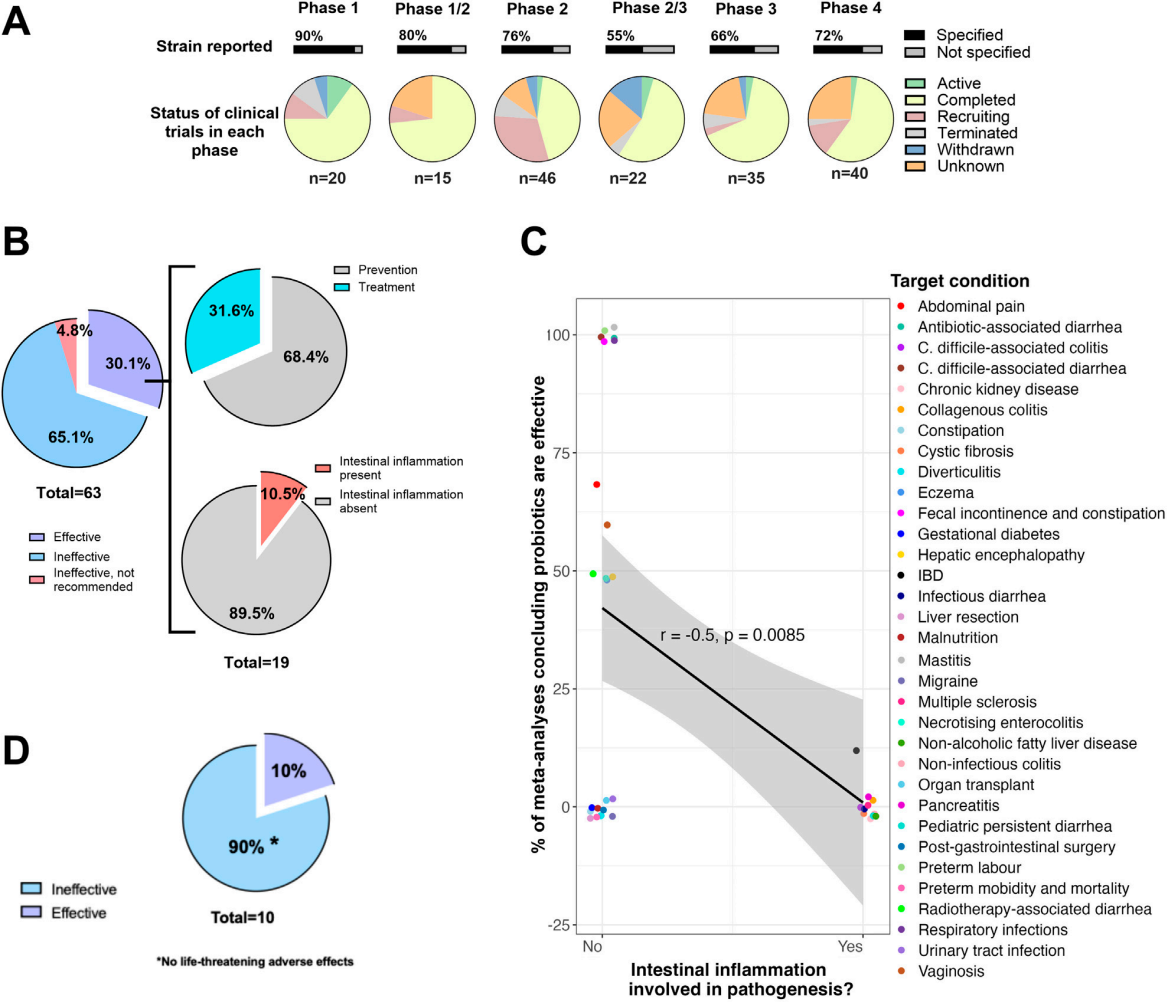
The ineffectiveness of LBPs is reflected in ambiguous clinical trials and meta-analyses. **(A)** Randomized controlled trials registered in clinicaltrials.gov for *Bifidobacterium* spp., showing the inconsistent trackability through clinical phases 1 to 4, and the lack of detailed reporting on the *Bifidobacterium* type strains used and the status of the studies in each phase. **(B)** Pie charts showing the conclusions of 63 meta-analyses retrieved from Cochrane Library, on the effect of probiotics (no strain distinction) on disease treatment and/or prevention, highlighting that the few studies with positive effects are associated with a lack of underlying intestinal inflammation. **(C)** The presence of intestinal inflammation in the target health condition is negatively correlated with the percentage of meta-analyses concluding probiotics are effective. Due to the non-normal distribution of the data, a Mann-Whitney U Test was used to test for significant differences between the 2 groups, and the effect size is determined using the Rank biserial correlation test. **(D)** Pie chart showing the efficacy of probiotics in IBD meta-analyses. Of the 63 meta-analyses identified, 10 meta-analyses specifically investigated IBD. No life-threatening adverse effects were reported. Authors AV and HC searched ClinicalTrials.gov for clinical trials using keywords like “Bifidobacteria.” They reviewed details on LBP strains, conditions studied, and trial statuses. Similarly, the authors AV and HC systematically retrieved all Cochrane reviews related to the keyword “live biotherapeutic product” (0 results) or “probiotic” from the Cochrane Library (https://www.cochranelibrary.com/). The researchers verified the quality of the results and extracted the authors’ conclusions from each meta-analysis. Figure 1 was created with GraphPad Prism and R.

Severe ambiguity surrounding the effectiveness of probiotics in clinical trials led us to question whether intestinal inflammation itself might act as a barrier to success in disease populations. However, before addressing this biological constraint, we must first acknowledge the challenges arising from the absence of a stringent regulatory framework for probiotics. These challenges arose because industry players approached regulatory approvals as dietary supplements instead of rigorous trials like those for drugs, given the cost, effort, and required expertise. The consequence of this was evident when we reviewed over 1,700 clinical studies on probiotics testing *Lactobacillus* spp. and *Bifidobacterium* spp. Revealing notable gaps in the reported data and a lack of rigor in trial design. Despite retrieving 581 clinical trials for Bifidobacteria (clinicaltrial.gov), only 31% specified essential information such as the clinical phase under investigation (Figure 1A). The lack of strain-level identification was striking, and tracing studies through all stages, from phase 1 to phase 4, proved nearly impossible. For example, of the 35 phase 3 studies identified, only 12 studies specified the strain of bacteria being used (drug class), and although 23 studies had a “completed” status, their results were not available, revealing a well-known publication bias for positive findings.

Given these data gaps, we turned to meta-analyses for clarity, anticipating that they would meet the benchmark of methodological rigor. Of the 63 meta-analyses found in the Cochrane Library (Supplementary Table S1), only 22 provided conclusive results, while 41 highlighted the heterogeneity of the findings, which statistically hindered the authors from reaching conclusions, leading to our classification as ineffective outcomes. It is important to note that 4% of analyses did not recommend the use of probiotics for the target disease, with one meta-analysis pinpointing harmful side effects (Supplementary Table S1), supporting that probiotics are not always safe in every patient population. Most strikingly, among the 30% of meta-analyses that reported LBPs to be effective, the overwhelming majority (89.5%) were conducted in populations without underlying intestinal inflammation (Figure 1B). These findings suggest that while LBPs may exhibit limited efficacy in treating active disease, more favorable effects in contexts where inflammation is absent or minimal could be apparent. This pattern was evident in studies on bacterial vaginosis, where 60% of meta-analyses demonstrated a positive clinical effect, as well as in disease-prevention trials involving otherwise healthy participants, where 68% of meta-analyses reported efficacy (Figure 1B). A rank-biserial correlation test supported the observation that the presence of intestinal inflammation negatively correlated with clinical outcomes, whereas the lack of intestinal inflammation correlated with positive clinical outcomes (Figure 1C). A single meta-analysis (including 7 studies) concluded that probiotics were clinically effective for inflammatory bowel disease (IBD) and may slightly improve the induction of remission. However, the authors reported very low-quality evidence of included studies and remarked on insufficient study information, failure to define remission, lack of blinding of participants, incomplete and selective reporting, poor quality of data and bias across the studies, in addition to no specific probiotic across studies (Kaur et al., 2020). Despite one positive analysis, approximately 90% of Cochrane meta-analyses conclude that probiotics, including those with LBP claims, lack clinical efficacy in treating IBD, a disease with chronic intestinal inflammation (Figure 1D). Given the limited efficacy of probiotics and LBPs in inflammatory disease contexts, intestinal inflammation appears to be a key biological barrier that undermines their therapeutic potential.

To explore further the observation that inflammation is a barrier to probiotic efficacy, we focused on the clinical evidence for *Escherichia coli* Nissle 1917 (EcN) treating ulcerative colitis (UC), a form of IBD. EcN is an extensively studied probiotic but has been tested in several clinical trials like it were an LBP. Of the 10 clinical trials reported, 6 focused on maintenance of remission, with the others testing induction of remission in active UC patients often comparing or adjunct to the frontline therapy, 5-aminosalicylic acid (5-ASA; Table 1). A meta-analysis including 5 of these trials concluded EcN is ineffective at inducing remission in patients with active UC, and any therapeutic benefit appears restricted to remission maintenance (Losurdo et al., 2015) (Table 1). This means that the more inflamed the gut environment is, like in active UC, the less likely EcN can confer any clinical benefit. A plausible explanation is EcN’s poor ability to persist and thus colonize the inflamed intestine. Still, to be effective even in remission, EcN still requires continuous high-dose daily administration with relapse rates between 16-45% and >60% 6 months post-cessation (Table 1). This is supported by data revealing EcN detectability drops precipitously post-cessation (Joeres-Nguyen-Xuan et al., 2010), observed in adults (Tannock et al., 2011) as well as infants (Cukrowska et al., 2002).

**TABLE 1:** Clinical trials and meta-analysis involving EcN for UC treatment.

Study	Intention of intervention	Sample size (n/N)	Daily dose^a^ (format)	Pre-treatment or additive therapy	Treatment comparison	Outcome^b^	Primary/Secondary endpoint	Result of the intervention	Weaknesses
^c^Matthes H. et al. BMC Complement Altern Med. 2010	To induce remission	70/90; 24 for 40 mL dose; 23 for 20 mL dose; 23 for 10 mL dose	40, 20 or 10 mL x 10^8 (enama)	NA	EcN vs. Placebo	−/+	DAI ≤2/time to remission, DAI = 0, histological healing	No differences in EcN vs. Placebo (ITT p = 0.4430); relapse rate in placebo 35% compared to 43.5%, 47.8%, and 36.4% remission in EcN 40 mL, 20 mL and 10 mL, respectively. Suggested dose-dependent efficacy in inducing remission (PP, p = 0.0443)	EcN dosage-dependency identified in PP was not observed in the ITT
^c^Petersen AM. et al. J Crohns Colitis. 2014	To induce remission	50/100; 25 in Ciprofloxacin/EcN; 25 in Placebo/EcN	5–50 × 10^9 (capsule)	5-ASA (≥64%) and/or thiopurines (≤32%)	Placebo/Placebo, Ciprof/Placebo, Placebo/EcN, and Ciprof/EcN (1 week/7 weeks)	−	CAI ≤4 (CRP and hemoglobin levels)/number of patients withdrawn from the study	No benefit in EcN use as an additional therapy to conventional treatment; a greater % of patients in the placebo group reached remission (P < 0.02) with remission rates of 80% for placebo/placebo, 72% for Cipro/placebo, 60% Cipro/EcN, and 41% placebo/EcN	Standard therapies were allowed to be modified by physicians throughout the study
Mirsepsai-Lauridsen HC. et al. Sci Rep. 2016	To induce remission	50/100, 25 in Ciprofloxacin/EcN; 25 in Placebo/EcN	5–50 × 10^9 (capsule)	Conventional therapies, except systemic steroids or TNF-α inhibitors	Placebo/Placebo, Ciprof/Placebo, Placebo/EcN, and Ciprof/EcN	−	Fecal calprotectin <200 mg/kg/colonization with *E. coli* B2	Reduction of fecal calprotectin was not superior than results in the placebo group (P < 0.05); remission rates of 61% for placebo/placebo, 57% for Cipro/placebo, 41% Cipro/EcN, and 18% placebo/EcN	
Park SK. et al. Korean J Gastroenterol. 2022	To induce remission	58/133	5 × 10^9 (capsule)	5-ASA	EcN and 5-ASA vs. placebo and 5-ASA	−/+	IBDQ >16/Mayo score decrease of 3 points, for clinical remission and 1 point for endoscopic	No differences in patients reaching primary endpoint (ITT, p = 0.86); 47% for placebo and 44.8% for EcN; secondary endpoints, including clinical remission and response rates, were better for EcN group	Did not specify 5-ASA dosage (merely mentioned that the dosage was stable throughout the trial)
^c^Rembacken BJ. et al., Lancet. 1999	To induce and to maintain remission	57/116	5 × 10^10 (capsule)	1 week of gentamicin and 2 weeks of hydrocortisone acetate enema	EcN vs. 5-ASA	+	Maintenance: Time to relapse and rate of relapse/Induction: rate and time to remission	Efficacy in maintaining remission after exacerbation of UC comparable to 5-ASA; relapse rate 73% in 5-ASA and 67% in EcN (significant equivalence p = 0.0059); remission induced in 75% in 5-ASA and 68% in EcN (significant equivalence p = 0.0508)	The statistics performed barely met the equivalence margin cut-off set by the authors (but was still able to statistically show that EcN was equivalent to 5-ASA)
^c^Kruis W. et al. Aliment Pharmacol Ther 1997	To maintain remission	50/120	5 × 10^10 (capsule)	NA	EcN vs. 5-ASA	+	Compare clinical remission (≤4 CAI)/compare relapse rates, relapse-free times and global assessment	Efficacy in maintaining remission and preventing relapse was comparable to 5-ASA (ITT p = 0.12); relapse rate 11.3% in 5-ASA and 16% in EcN	
^c^Kruis W. et al. Gut. 2004	To maintain remission	162/327	5–50 × 10^9 (capsule)	NA	EcN vs. 5-ASA	+/−	Relapse prevention; relapse identified when CAI>6 or +3 points in CAI or endoscopic index >4/general wellbeing, and calculation of a quality of life index	Efficacy and safety in maintaining remission comparable to 5-ASA; relapse rate 37% in 5-ASA and 45.1% in EcN (ITT significant equivalence, p = 0.013); no significant differences observed in secondary endpoints	
^c^ ^,^ ^d^Henker J. et al. Z Gastroenterol. 2008	To maintain remission	24/34	At least 5 × 10^10 (capsule)	NA	EcN vs. 5-ASA	+	Relapse prevention/quality of life index	Efficacy in maintaining remission comparable to 5-ASA; relapse rate of 30% in 5-ASA and 25% in EcN, no statistical analysis reported	No statistics were reported in the study
Oh GM. et al. Korean J Gastroenterol. 2021	To maintain remission	94^e^	5 × 10^9 (capsule)	90.4% on 5-ASA ±30.9% in thiopurine	None^e^	−/+	Changes in fecal calprotectin/Mayo score, body weight, BMI, and hemoglobin	IBDQ scores no different between EcN and placebo groups (PP p = 1, ITT p = 0.86); effective for symptom reduction when used with other therapies (P = 0.031), but not for fecal calprotectin (P = 0.653)^e^	Retrospective study
Bodini G. et al. J Gastrointestin. Liver Dis. 2023	To maintain remission	49/94^e^	First month 2 capsules; second month 1 capsule	31% on biologics, 8% on immuno-suppressants and 4% on steroids	None^e^	−/+	EcN’s effect on Fecal calprotectin values in clinical remission	Effective in maintaining remission after 2 months of concomitant treatment (P < 0.005); >60% patients relapsed post 6 months cessation^e^ ^,^ ^f^	No prospective control group
^g^Losurdo G. et al. J Gastrointestin. Liver Dis. 2015								EcN is equivalent to 5-ASA in remission maintenance, but EcN is not superior to placebo in inducing remission	

CAI, clinical activity index; DAI, disease activity index; IBDQ, inflammatory bowel disease questionnaire; ITT, intention to treat; NA, not available; PP, per protocol.

^a^ Most studies reported that each capsule contained 2.5 × 10^9, and patients received two capsules/day.

^b^ (+) Better than placebo or same as 5-ASA, for primary endpoint; (−) Same as placebo or inferior to 5-ASA, for primary endpoint; (−/+) For negative primary endpoint but positive for relevant secondary endpoint or *vice versa*.

^c^ Study included in the meta-analysis by Losurdo G et al. J Gastrointestin Liver Dis. 2015.

^d^ All the studies focused on adults, except Henker et al., 2008, which only included youth aged 11 to 18.

^e^ Retrospective study. If a control group was included, it was a matched no-prospective control group.

^f^ As stated in the correspondence letter to the editor (Pasta et al., 2024).

^g^ Only meta-analysis identified for EcN in UC.

These data highlight a systemic challenge in microbial therapy, the inability to overcome colonization resistance and achieve durable engraftment, particularly under inflammatory conditions. This limitation explains why the American Gastroenterology Association (AGA) and the European Crohn’s and Colitis Organization (ECCO) do not endorse any probiotic, including EcN, as a primary therapy for IBD (Su et al., 2020; Harbord et al., 2017). EcN exemplifies this broader issue affecting microbiome-based therapeutics, including transient colonization, high dosing requirements, and limited efficacy during active disease. Still, EcN has been recognized as a potential complementary medicine to maintain remission in UC patients by ECCO but not AGA, given its general safety profile and potential to add benefit (Su et al., 2020; Torres et al., 2019).

## The translational roadblocks to live biotherapeutic product success

Colonization is a major challenge that jeopardizes the success of any microbial therapy, probiotics or LBPs. Indeed, several research groups have demonstrated that probiotics show poor engraftment into the host’s microbial ecosystem (Zmora et al., 2018). While ecological theories of colonization resistance, where LBPs must compete with native gut microbes for successful niche establishment, are well known, other hurdles arise even earlier during the manufacturing and formulation stages. First, the microbe must remain viable throughout the production process and remain stable during storage. Ideally, LBPs should have a long shelf life at room temperature without loss of potency. This maintenance of long shelf life and stability at ambient temperatures can be achieved through means like microencapsulation. However, from our review of meta-analyses (Supplementary Table S1), we found that the vast majority of studies did not report the use of probiotic formulations, including microencapsulation, needed to extend shelf life. Second, the viable product must then withstand the harsh gastric acid exposure when taken by the subject, to reach the small or large intestine, the final destination to exert its beneficial effects. Enteric-coated capsule technologies designed to be acid-resistant can significantly improve the delivery of intact microbes to the intestine. Other novel solutions include the use of hydrogels that allow the formation of probiotic microcolonies, offering protection and favouring probiotic bacterium behaviour for increased colonization (Liu et al., 2025). Surprisingly, very few studies reported the use of enteric-coated capsules for the delivery of the microbial therapy (Supplementary Table S1).

Another challenge is the need for mitigation strategies against adverse microbial adaptations, as microbes can rapidly evolve and mutate within their host. Reports have identified mutations linked to antibiotic-resistance genes in probiotics isolated from patients with bacteremia (Yelin et al., 2019). This has been observed in patients treated simultaneously with antibiotics and probiotics, where the latter supported the growth of vancomycin-resistant strains (Montassier et al., 2021). Furthermore, since LBPs can modulate the gut microbiome as part of their therapeutic mechanism, other vital considerations, such as inter-individual microbiome differences, must be accounted for while selecting the most suitable LBP for the patient. Such appreciation for inter-individual variabilities is increasingly relevant as the age of “personalized medicine”, or individualized treatment approaches, has taken center stage.

Finally, the loose regulatory framework used for probiotics has been permissive for many health claims not supported by high-quality evidence. Moreover, there is a lack of consistency in global regulatory frameworks, as evidenced by differences between the FDA in contrast to the Australian Therapeutic Goods Administration, which has tailored and updated guidelines for the quality of LBPs. The scope of microbiome-based therapies is moving towards the use of the LBPs framework instead of general probiotics, but this could be as messy and as unreliable as probiotics without a harmonized approach.

## Turning microbes into functional medicine

### Is bioengineering the future?

What is needed to turn microbes into functional medicines that are reliable and clinically meaningful for the plethora of clinical challenges we face? Certainly, LBPs may need to be tailored to the patient, considering their host-associated factors, including the immunological status and their individualized microbiomes. This specific approach may require bioengineering microbes to target a specific desired effect. For example, engineered LBPs like *Lactococcus lactis* secreting IL-10 or diverse strains of *Lactobacillus* spp. Secreting superoxide dismutase both showed promising results in pre-clinical models of colitis (del Carmen et al., 2014; Watterlot et al., 2010), but still failed to translate into clinically relevant interventions in clinical trials (Clinicaltrials, 2009). A trailblazing company, Synlogic, took their LBP SYNB 1934, an engineered variant of EcN for treating phenylketonuria, from pre-clinical studies to clinical phase 3 (Clinicaltrials, 2023). Despite the initial promise, the trial was terminated because the study was unlikely to meet the primary endpoint (Synlogic, 2024). Nevertheless, this trial lights the path towards the potential for rationalized bioengineering of LBPs.

**FIGURE 2:**
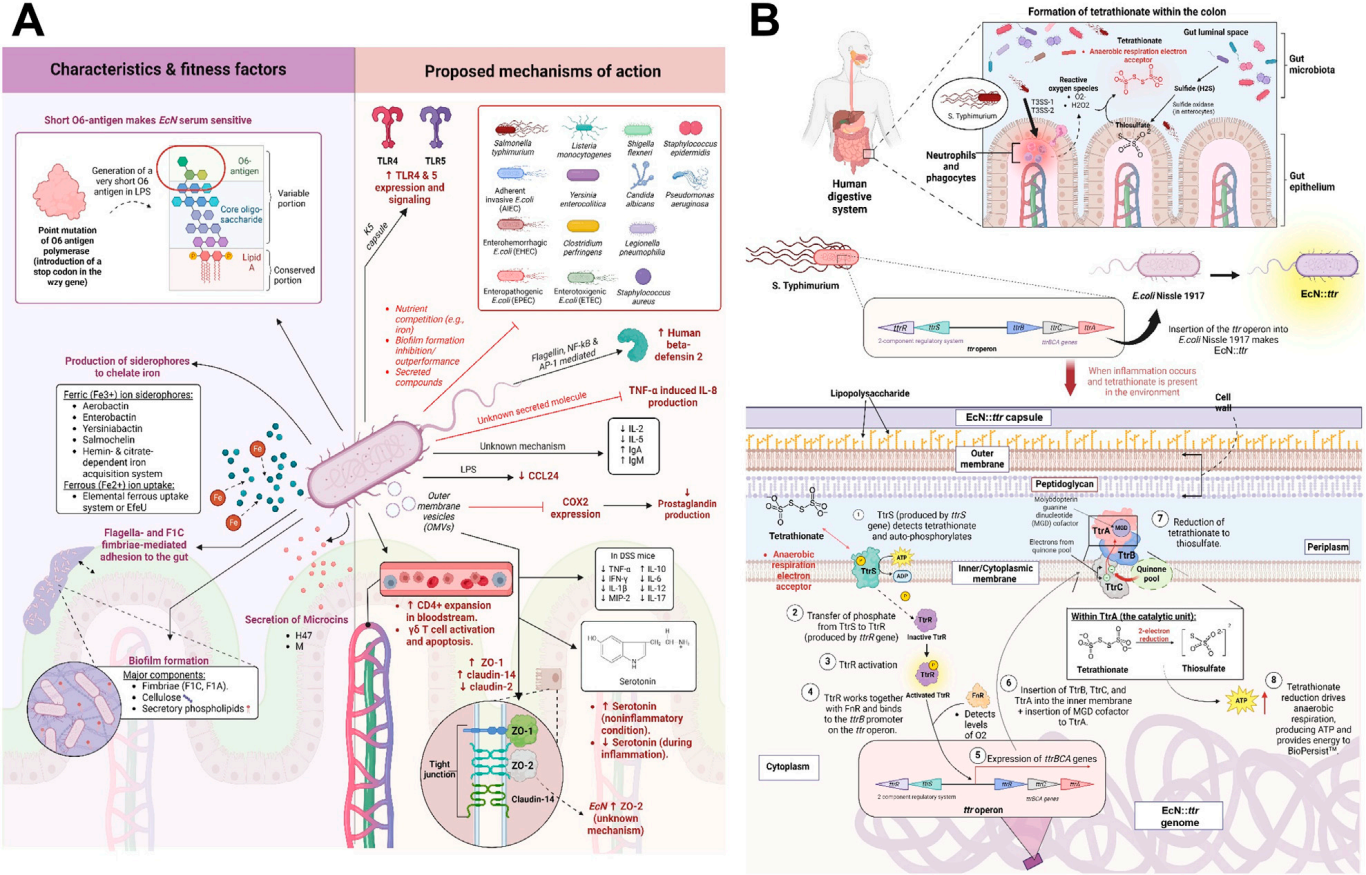
Engineering *E. coli* Nissle 1917 as an example of a rationalized LBP. **(A)** Summary of the characteristics or fitness factors and proposed mechanisms of action of the LBP *E. coli* Nissle 1917 (EcN; serotype O6:K5:H1); Phenotypically, *E. coli* Nissle 1917 (EcN) possesses an atypical O6 antigen with only one saccharide unit as a result of a point mutation in the O6 antigen polymerase *wzy* gene, rendering EcN serum-sensitive as the O antigen is critical for bacterial virulence (Grozdanov et al., 2002; Sonnenborn and Schulze, 2009). EcN is also capable of forming biofilms, which consists of F1C fimbriae, F1A fimbriae, cellulose, and secretory phospholipids (Sonnenborn and Schulze, 2009). EcN is notable for its ability to defend against a variety of pathogenic (or potentially pathogenic) organisms through means such as nutrient competition, outperformance/inhibition of biofilm formation, and (to a certain extent) secretion of microcins H47 and M (Sonnenborn and Schulze, 2009; Fang et al., 2018; Hafez et al., 2010; Ma et al., 2023). EcN K5 capsule and flagellin can also enhance toll-like receptor (TLR)-4 and −5 as well as human β-defensin 2 production (respectively), both of which can enhance pathogen defense and gut homeostasis (Hafez et al., 2010). EcN also possesses immunomodulating properties. Through unknown mechanisms, EcN can prevent tumor necrosis factor (TNF)-α induced interleukin (IL)-8 production in HCT15 cells, reduce the levels of IL-2 and IL-5, and increase the levels of immunoglobulin (Ig) A and M (Sonnenborn and Schulze, 2009). EcN lipopolysaccharide (LPS) has been shown to downregulate chemokine CCL24 expression (and thus, inflammation) in human peripheral blood mononuclear cells (Güttsches et al., 2012). EcN increases bloodstream naïve and memory CD4^+^ T cells (without influencing gut epithelial T cell populations) and regulates the activation/progression and apoptosis of γδ T cells, enabling an immune response adequately strong to target inflammation-triggering agents (e.g., bacterial antigens) while controlling the extent of gut inflammation (Sonnenborn and Schulze, 2009). EcN can also influence gut metabolites and epithelial barrier integrity. Serotonin, which is involved in gut motility and signaling, was previously shown to be elevated by EcN under non-inflammatory conditions through an unknown mechanism (Nzakizwanayo et al., 2015); later experiments showed that EcN outer membrane vesicles (OMVs) can modulate serotonin levels, particularly during inflammation (Olivo-Martínez et al., 2024). In mice with dextran sodium sulfate (DSS)-induced colitis, OMVs reduce the expression of cyclooxygenase 2 (COX2), an enzyme that synthesizes prostaglandin, and modulates cytokine levels (Olivo-Martínez et al., 2024). EcN OMVs have also been shown to increase the expression of tight junction (TJ) proteins zonula occludens (ZO)-1 and claudin-14 while reducing claudin-2 expression, all of which strengthen the gut barrier (Olivo-Martínez et al., 2024). EcN can also increase the expression of another TJ protein called ZO-2 (Olivo-Martínez et al., 2024), though the specific mechanism is unclear. **(B)** Summary of EcN::*ttr* mechanism of persistence; EcN::*ttr* is created by bio-engineering EcN with a *Salmonella typhimurium-*derived operon called the *ttr* operon, which consists of five genes: *ttrS, ttrR, ttrB, ttrC,* and *ttrA* (Price-Carter et al., 2001). The *ttr* operon-derived enzyme tetrathionate reductase enables *S. typhimurium* to anaerobically respire using tetrathionate, an inflammatory metabolite produced from thiosulfate and reactive oxygen species (that infiltrates into the gut during inflammation). Though it remains elusive how EcN can specifically detect tetrathionate, tetrathionate presence initiates a cascade of reactions within EcN::*ttr*, starting with the autophosphorylation of the membrane-bound protein TtrS followed by the activation of the TtrR protein through a phosphate translation event (Price-Carter et al., 2001). Activated TtrR induces the transcription and assembly of the tetrathionate reductase enzyme, which catalyzes the reduction of tetrathionate into thiosulfate (Price-Carter et al., 2001). Tetrathionate reduction drives anaerobic respiration and boosts ATP production (Price-Carter et al., 2001), allowing EcN::*ttr* to thrive within inflammatory conditions. Created in BioRender. Chiang, H. (2025) https://BioRender.com/rod4eh5.

A key takeaway from these translational failures is that the inflammatory environment in many chronic diseases may not be conductive for live microbial therapies to act effectively, resulting in diminished efficacy and inconsistent clinical outcomes. One approach to overcoming this challenge is to genetically modify the probiotic to be tolerant or have a survival advantage in the inflamed environment. Our group has begun to address this by bioengineering EcN to have a fitness advantage during inflammation. Although EcN has been shown to promote multiple intestinal benefits in mostly rodent or tissue culture studies (Figure 2A), clinical studies in IBD patients have indicated no measurable and consistent differences in effectiveness when compared to placebo or even first-line therapy during active disease (Table 1). Based on the observation that intestinal inflammation is correlated with the lack of clinical efficacy of probiotics (Figure 1), we hypothesized that the intestinal inflammation prevents enough engraftment during colitis, limiting EcN’s ability to elicit its beneficial effects (Gibson et al., 2018). To overcome EcN’s colonization limitations during inflammation, we introduced genetic tools to support its persistence and expansion during colitis (Figure 2B), resulting in a more consistent protection, as we have shown in pre-clinical models (Verdugo-Meza et al., 2024). Understanding how EcN::*ttr* translates into clinical outcomes will be key in the next-generation of bioengineered LBPs.

Bioengineered LBPs may provide promises of the next-generation of microbial medicines, but several safety challenges must be addressed. For example, bioengineered LBP design should account for known risks like horizontal gene transfer, environmental biocontainment, and growth control in the patient’s ecosystem with the addition of kill switches (Rottinghaus et al., 2022). More studies are needed to know if these are real or just perceived risks to understand if we must address these challenges to support the safe use of engineered LBPs. Potential risks can be addressed by adhering to FAO/WHO guidelines for characterizing administered microbes, including assessing antibiotic resistance and metabolic activity (such as D-lactate production or bile salt deconjugation), testing for toxin production, and checking for hemolytic activity. It is important to evaluate genetic stability through whole genome sequencing, test for virulence factors, and ideally evaluate horizontal gene transfer capacity, including phage sensitivity. By employing this approach, the negative ecological impact on the host’s microbiome and side effects such as systemic microbial translocation and abnormal immune responses in vulnerable patient populations may be reduced.

### Strategies to enhance the efficacy of live biotherapeutic products

Even if bioengineered or otherwise optimized LBPs ultimately demonstrate clinical efficacy, significant manufacturing challenges remain. Chief among these is ensuring that LBPs reach the intestine intact and viable. While simple enteric-coated capsules can protect against gastric acid degradation, microencapsulation should become standard practice in the field to maintain viability prior to the patient consuming the product. This technique has long been shown to preserve the stability of lyophilized microbes during storage and to enhance overall product viability. Microencapsulation, often employing alginate-based matrices supplemented with cryoprotectants, provides an effective means of stabilizing bacterial formulations and extending shelf life (Razavi et al., 2021). Moreover, multi-layered encapsulation systems can offer additional protection while enabling controlled release of viable microbes throughout the small and large intestine. Beyond these established approaches, next-generation encapsulation strategies, such as engineered polymer “cages,” are being developed to achieve spatially targeted and temporally controlled microbial release along the gastrointestinal tract (Finbloom et al., 2023).

Finally, there is an imperative need for the regulatory systems built exclusively for LBPs, including bioengineered LBPs. This regulatory system should standardize how we evaluate the health claims for LBPs. An example of this could be guidelines for clinical trials that account for the survival, engraftment and clearance of the evaluated LBP. In addition, given that diet exerts a major influence on microbial composition, function and metabolite production, reliable assessment of patients’ dietary status could become routine in clinical trials testing LBPs in patient populations. If industry moves towards employing already well-researched methods of microencapsulation of LBPs, then we need to assume that the dose of the drug is the dose delivered into the GI tract. This may mean dose escalation challenges will be important to consider, since a therapeutic dose may in fact be far less than typically used with most current products. This could be critical for susceptible populations like pre-term infants with necrotizing enterocolitis, shown to be susceptible to sepsis with probiotic treatment (Chiang et al., 2021). Lastly, the regulatory system should be adaptable as we keep gaining knowledge about host-microbiome interactions in health and disease.

## Translating promise into practice

Over the past 5 years, investments in microbiome-based therapeutics have soared, reflecting immense optimism in their potential. Yet, stakeholders have been largely disappointed by underwhelming clinical outcomes. The initial wave of investor enthusiasm is now confronting a reality check, as efficacy results remain inconsistent and frequently disappointing (Zamecnik, 2023). Notable examples include consortia LBPs for IBD treatment, such as SER-287 (Seres Therapeutics) and VE202 (Vedanta Biosciences). Both advanced to Phase 2 clinical trials but were ultimately terminated after failing to meet primary endpoints. Despite these setbacks, the field continues to evolve rapidly driven by advances in engineered LBPs, precision microbiome approaches integrated with predictive modelling (Markets Ra, 2025; Eisenstein, 2020). Looking ahead, meaningful progress will depend on developing microbial strains or consortia capable of durable colonization and mechanistic alignment with disease pathophysiology.

By addressing key limitations in clinical evidence, quality control, and patient stratification, the field may finally achieve targeted and reproducible therapeutic outcomes. This may include rational genetic modifications to enhance LBP function in specific disease contexts and integrating advanced formulation technologies to ensure both targeted delivery and shelf-life stability. For many chronic inflammatory diseases, where treatment options remain limited to broad immunosuppression, LBPs offer a transformative paradigm, one that leverages their immunomodulatory properties and host-microbe synergy to restore health. Implementing rational yet practical strategies to enhance clinical effectiveness may ultimately allow LBPs to fulfill their long-promised therapeutic potential.

## Data Availability

The original contributions presented in the study are included in the article/Supplementary Material, further inquiries can be directed to the corresponding author.

## References

[B38] Bland C. M. Love B. L. Jones B. M. (2025). Human microbiome: Impact of newly approved treatments on C. difficile infection. Am. J. Health Syst. Pharm. 82, 174–183. 39230353 10.1093/ajhp/zxae249

[B1] Chiang M. C. Chen C. L. Feng Y. Chen C. C. Lien R. Chiu C. H. (2021). Lactobacillus rhamnosus sepsis associated with probiotic therapy in an extremely preterm infant: pathogenesis and a review for clinicians. J. Microbiol. Immunol. Infect. 54, 575–580. 10.1016/j.jmii.2020.03.029 32307246

[B2] Clinicaltrials (2009). A phase 2a randomized, placebo-controlled, double-blind, multi-center dose escalation study, to evaluate the safety, tolerability, pharmacodynamics and efficacy of AG011, in subjects with moderately active ulcerative colitis. Available online at: https://clinicaltrials.gov/study/NCT00729872.

[B3] Clinicaltrials (2023). A phase 3, double-blind, placebo-controlled, randomized withdrawal study to evaluate the efficacy and safety of SYNB1934 in patients with PKU (SYNPHENY-3).

[B4] Cukrowska B. LodInová-ZádnIková R. Enders C. Sonnenborn U. Schulze J. Tlaskalová-Hogenová H. (2002). Specific proliferative and antibody responses of premature infants to intestinal colonization with nonpathogenic probiotic E. coli strain nissle 1917. Scand. J. Immunol. 55 (2), 204–209. 10.1046/j.1365-3083.2002.01005.x 11896937

[B5] del Carmen S. Martín Rosique R. Fau - Saraiva T. Saraiva T. Fau - Zurita-Turk M. Zurita-Turk M. (2014). Protective effects of lactococci strains delivering either IL-10 protein or cDNA in a TNBS-induced chronic colitis model. J. Clin. Gastroenterol. 48, S12–S17. 10.1097/MCG.0000000000000235 25291117

[B6] Eisenstein M. (2020). Early investments powering the ascent of microbiome therapeutics biopharma dealmakers. Nature. 10.1038/d43747-020-01178-x

[B7] Fang K. Jin X. Hong S. H. (2018). Probiotic *Escherichia coli* inhibits biofilm formation of pathogenic *E. coli via* extracellular activity of DegP. Sci. Rep. 8 (1), 4939. 10.1038/s41598-018-23180-1 29563542 PMC5862908

[B8] Finbloom J. A. Huynh C. Huang X. Desai T. A. (2023). Bioinspired nanotopographical design of drug delivery systems. Nat. Rev. Bioeng. 1 (2), 139–152. 10.1038/s44222-022-00010-8

[B9] Gibson D. L. Godovannyi A. Gill S. (2018). Probiotic compositions and uses thereof. WO2007140621A1.

[B10] Grozdanov L. Zähringer U. Blum-Oehler G. Brade L. Henne A. Knirel Y. A. (2002). A single nucleotide exchange in the *wzy* gene is responsible for the semirough O6 lipopolysaccharide phenotype and serum sensitivity of *Escherichia coli* strain nissle 1917. J. Bacteriol. 184 (21), 5912–5925. 10.1128/JB.184.21.5912-5925.2002 12374825 PMC135379

[B11] Güttsches A.-K. Löseke S. Zähringer U. Sonnenborn U. Enders C. Gatermann S. (2012). Anti-inflammatory modulation of immune response by probiotic *Escherichia coli* nissle 1917 in human blood mononuclear cells. Innate Immun. 18 (2), 204–216. 10.1177/1753425910396251 21382908

[B12] Hafez M. Hayes K. Goldrick M. Grencis R. K. Roberts I. S. (2010). The K5 capsule of *Escherichia coli* strain nissle 1917 is important in stimulating expression of toll-like receptor 5, CD14, MyD88, and TRIF together with the induction of Interleukin-8 expression *via* the mitogen-activated protein kinase pathway in epithelial cells. Infect. Immun. 78 (5), 2153–2162. 10.1128/IAI.01406-09 20145095 PMC2863526

[B13] Harbord M. Eliakim R. Bettenworth D. Karmiris K. Katsanos K. Kopylov U. (2017). Third European evidence-based consensus on diagnosis and management of ulcerative colitis. Part 2: current management. J. Crohns Colitis 11 (7), 769–784. 10.1093/ecco-jcc/jjx009 28513805

[B37] Heavey M. K. Durmusoglu D. Crook N. Anselmo A. C. (2022). Discovery and delivery strategies for engineered live biotherapeutic products. Trends Biotechnol. 40, 354–369. 10.1016/j.tibtech.2021.08.002 34481657 PMC8831446

[B39] Henker J. Müller S. Laass M. W. Schreiner A. Schulze J. (2008). Probiotic Escherichia coli Nissle 1917 (EcN) for successful remission maintenance of ulcerative colitis in children and adolescents: an open-label pilot study. Z. Gastroenterol. 46 (9), 874–875. 10.1055/s-2008-1027463 18810672

[B14] Joeres-Nguyen-Xuan T. H. Boehm S. K. Joeres L. Schulze J. Kruis W. (2010). Survival of the probiotic *Escherichia coli* nissle 1917 (EcN) in the gastrointestinal tract given in combination with oral mesalamine to healthy volunteers. Inflamm. Bowel Dis. 16 (2), 256–262. 10.1002/ibd.21042 19637333

[B15] Kaur L. Gordon M. Baines P. A. Iheozor-Ejiofor Z. Sinopoulou V. Akobeng A. K. (2020). Probiotics for induction of remission in ulcerative colitis. Cochrane Database Syst. Rev. 3. 10.1002/14651858.CD005573.pub3 32128795 PMC7059959

[B16] Liu H. Chen Z. Lin Q. Chen Y. Hong L. Zhong J. (2025). A multicellular self-organized probiotic platform for oral delivery enhances intestinal colonization. Nat. Commun. 16 (1), 7060. 10.1038/s41467-025-62349-x 40750778 PMC12317005

[B17] Losurdo G. Iannone A. Contaldo A. Ierardi E. Di Leo A. Principi M. (2015). *Escherichia coli* nissle 1917 in ulcerative colitis treatment: systematic review and meta-analysis. J. Gastrointestin Liver Dis. 24 (4), 499–505. 10.15403/jgld.2014.1121.244.ecn 26697577

[B18] Ma Y. Fu W. Hong B. Wang X. Jiang S. Wang J. (2023). Antibacterial MccM as the major microcin in *Escherichia coli* nissle 1917 against pathogenic enterobacteria. IJMS 24 (14), 11688. 10.3390/ijms241411688 37511446 PMC10380612

[B19] Markets Ra (2025). Microbiome therapeutics market revenues to double by 2030 - key opportunities lie in live-biotherapeutics, personalized medicine, and digital health integrations GLOBE NEWSWIRE. Available online at: https://www.globenewswire.com/news-release/2025/09/02/3142416/28124/en/Microbiome-Therapeutics-Market-Revenues-to-Double-by-2030-Key-Opportunities-Lie-in-Live-biotherapeutics-Personalized-Medicine-and-Digital-Health-Integrations.html.

[B20] Montassier E. A.-O. Valdés-Mas R. A.-O. Batard E. Zmora N. Dori-Bachash M. Suez J. A.-O. X. (2021). Probiotics impact the antibiotic resistance gene reservoir along the human GI tract in a person-specific and antibiotic-dependent manner. Nat. Microbiol. 6 (8), 1043–1054. 10.1038/s41564-021-00920-0 34226711 PMC8318886

[B21] Nzakizwanayo J. Dedi C. Standen G. Macfarlane W. M. Patel B. A. Jones B. V. (2015). *Escherichia coli* nissle 1917 enhances bioavailability of serotonin in gut tissues through modulation of synthesis and clearance. Sci. Rep. 5 (1), 17324. 10.1038/srep17324 26616662 PMC4663480

[B22] Olivo-Martínez Y. Martínez-Ruiz S. Cordero-Alday C. Bosch M. Badia J. Baldoma L. (2024). Modulation of serotonin-related genes by extracellular vesicles of the probiotic *Escherichia coli* nissle 1917 in the Interleukin-1β-Induced inflammation model of intestinal epithelial cells. IJMS 25 (10), 5338. 10.3390/ijms25105338 38791376 PMC11121267

[B23] Pasta A. Calabrese F. Bodini G. (2024). Response to letter to editor on reduction of fecal calprotectin levels induced by a short course of Escherichia coli nissle is associated with a lower likelihood of disease flares in patients with ulcerative colitis in clinical remission. J. Gastrointestin. Liver Dis. 33 (2), 281. 10.15403/jgld-5660 38944863

[B24] Price-Carter M. Tingey J. Bobik T. A. Roth J. R. (2001). The alternative electron acceptor tetrathionate supports B12-dependent anaerobic growth of *Salmonella enterica* serovar typhimurium on ethanolamine or 1,2-propanediol. J. Bacteriol. 183 (8), 2463–2475. 10.1128/JB.183.8.2463-2475.2001 11274105 PMC95162

[B25] Razavi S. Janfaza S. Tasnim N. A.-O. Gibson D. L. Hoorfar M. A.-O. (2021). Nanomaterial-based encapsulation for controlled gastrointestinal delivery of viable probiotic bacteria. Nanoscale Adv. 3, 2699–2709. 10.1039/d0na00952k 36134186 PMC9419840

[B26] Rottinghaus A. G. Ferreiro A. Fishbein S. R. S. Dantas G. Moon T. S. (2022). Genetically stable CRISPR-Based kill switches for engineered microbes. Nat. Commun. 13 (1), 672. 10.1038/s41467-022-28163-5 35115506 PMC8813983

[B27] Sonnenborn U. Schulze J. (2009). The non-pathogenic *Escherichia coli* strain nissle 1917–features of a versatile probiotic. Microb. Ecol. Health Dis. 21 (3-4), 122–158. 10.3402/mehd.v21i3-4.7512

[B28] Su G. L. Ko C. W. Bercik P. Falck-Ytter Y. Sultan S. Weizman A. V. (2020). AGA clinical practice guidelines on the role of probiotics in the management of gastrointestinal disorders. Gastroenterology 159 (2), 697–705. 10.1053/j.gastro.2020.05.059 32531291

[B29] Synlogic (2024). Synlogic announces decision to discontinue Synpheny-3 study and provides corporate update. Available online at: https://investor.synlogictx.com/news-releases/news-release-details/synlogic-announces-decision-discontinue-synpheny-3-study-and#:∼:text=%22It%20is%20with%20a%20heavy,%2C%22%20said%20Aoife%20Brennan%20%2C%20M.B.

[B30] Tannock G. W. Tiong I. S. Priest P. Munro K. Taylor C. Richardson A. (2011). Testing probiotic strain *Escherichia coli* nissle 1917 (mutaflor) for its ability to reduce carriage of multidrug-resistant *E. coli* by elderly residents in long-term care facilities. J. Med. Microbiol. 60 (Pt 3), 366–370. 10.1099/jmm.0.025874-0 21127156

[B31] Torres J. Ellul P. Langhorst J. Mikocka-Walus A. Barreiro-de Acosta M. Basnayake C. (2019). European crohn's and colitis organisation topical review on complementary medicine and psychotherapy in inflammatory bowel disease. J. Crohns Colitis 13 (6), 673–685e. 10.1093/ecco-jcc/jjz051 30820529

[B32] Verdugo-Meza A. Gill S. K. Godovannyi A. Adur M. K. Barnett J. A. Estaki M. (2024). Bio-engineering a common probiotic to exploit colonic inflammation promotes reliable efficacy in translational models of colitis. bioRxiv. 10.1053/j.gastro.2026.04.007 42066862

[B33] Watterlot L. Rochat T. Fau - Sokol H. Sokol H. Fau - Cherbuy C. Cherbuy C. (2010). Intragastric administration of a superoxide dismutase-producing recombinant Lactobacillus casei BL23 strain attenuates DSS colitis in mice. Int. J. Food Microbiol. 144 (1), 35–41. 10.1016/j.ijfoodmicro.2010.03.037 20452077

[B34] Yelin I. A.-O. Flett K. B. Merakou C. Mehrotra P. Stam J. Snesrud E. (2019). Genomic and epidemiological evidence of bacterial transmission from probiotic capsule to blood in ICU patients. Nat. Med. 25 (11), 1728–1732. 10.1038/s41591-019-0626-9 31700189 PMC6980696

[B35] Zamecnik A. (2023). Is the microbiome therapy hype up for a reckoning? Pharmaceutical technology. Available online at: https://www.pharmaceutical-technology.com/features/is-the-microbiome-therapy-hype-up-for-a-reckoning/.

[B36] Zmora N. Zilberman-Schapira G. Suez J. Mor U. Dori-Bachash M. Bashiardes S. (2018). Personalized gut mucosal colonization resistance to empiric probiotics is associated with unique host and microbiome features. Cell 174 (6), 1388–405.e21. 10.1016/j.cell.2018.08.041 30193112

