# Supplementary material for: Not quite a cure yet: unlocking the unfulfilled promise of live biotherapeutics for disease treatment
Source: Front Pharmacol. 2025 Nov 5;16:1695976. doi: 10.3389/fphar.2025.1695976 (PMC12626851; doi:10.3389/fphar.2025.1695976)
Supplement: Supplementary file 1 [file DataSheet1.pdf]

Table 1. Summary of meta-analyses on the use of probiotics. Cochrane reviews were obtained from the Cochrane Library (accessed November, 2024-February 19, 2025, cochranelibrary.com), summarizing the gut inflammatory status (based on the target disease being treated) and the overall results for each meta-analysis.

Target condition	Underlying gut inflammation	Scope of treatment	Overall result	% of meta-analyses concluding probiotics are effective	% encapsulated packaging	% Gelatine capsules	Studies included	PubMed ID (PMID)
Abdominal pain	No	Prevention	Ineffective	66.7	0	0	6	30865287
	No	Prevention	Effective		38.89 (7/18)	0	18	36799531
	No	Treatment	Effective		38.46 (5/13)	0	13	28334433
Chronic kidney disease	Yes	Adjunct treatment	Ineffective	0.0	16.67 (5/30)	6.67 (2/30)	30	37870148
Colitis, *C. difficile*-associated	Yes	Treatment	Ineffective	0.0	n/a	n/a	4	18254055
Colitis, collagenous	Yes	Treatment	Ineffective	0.0	0	0	1	29127772
Colitis, non-infectious	Yes	Prevention	Ineffective	0.0	50 (1/2)	0	2	35470864
Constipation	No	Treatment	Ineffective	0.0	14.39 (2/14)	0	14	35349168
Cystic fibrosis	Yes	Adjunct treatment	Ineffective	0.0	16.67 (2/12)	16.67 (2/12)	12	31962375
Diarrhea	Yes	Treatment	Ineffective	0.0	0	0	4	23963712
Diarrhea, antibiotic-associated	No	Prevention	Effective	100.0	3.03 (1/33)	0	33	31039287

Diarrhoea, *C. difficile*-associated	Yes	Prevention	Effective	100.0	17.95 (7/39)	5.13 (2/39)	39	29257353
Infectious diarrhoea	Yes	Treatment	Ineffective	0.0	3.66 (3/82)	6.10 (5/82)	82	33295643
Diverticulitis	No	Prevention	Ineffective	0.0	50 (1/2)	0	2	28973845
Eczema	No	Treatment	Ineffective	50.0	2.56 (1/39)	0	39	30480774
	No	Prevention	Effective		n/a	n/a	5	17943912
Fecal incontinence and constipation	No	Adjunct treatment	Effective	100.0	0	0	5	39470206
Gestational diabetes	No	Treatment	Ineffective	0.0	11.11 (1/9)	22.22 (2/9)	9	32575163
	No	Prevention	Ineffective		n/a	n/a	n/a	32526091
	No	Prevention	**Ineffective, not recommended, harmful side-effect**		14.29 (1/7)	0	7	33870484
Hepatic encephalopathy	No	Prevention, adjunct treatment	Ineffective	50.0	0	50 (1/2)	2	29762873
	No	Prevention, adjunct treatment	Effective		9.52 (2/21)	9.52 (2/21)	21	28230908
Inflammatory bowel disease	Yes	Prevention	Ineffective	10.0	n/a	n/a	23?	19821389
	Yes	Treatment	Ineffective		n/a	n/a	1	38501688
	Yes	Treatment	Ineffective		0	0	5	31513295
	Yes	Treatment	Ineffective		0	1/2	2	30736095

	**Yes**	Treatment	Effective		**21.43 (3/14)**	**14.29 (2/14)**	**14**	**32128795**
	Yes	Treatment	Ineffective		8.33 (1/12)	1/12 (8.33)	12	32128794
	Yes	Treatment	Ineffective		0	50 (1/2)	2	32678465
	Yes	Prevention, treatment	Ineffective		14.29 (1/7)	14.29 (1/7)	7	31785173
	Yes	Treatment	Ineffective		0	50 (1/2)	2	35583095
	Yes	Treatment	**Ineffective, not recommended**		14.29 (1/7)	n/a	7	17054217
Liver resection	No	Adjunct treatment	Ineffective	0.0	n/a	n/a	n/a	22071832
Malnutrition	No	Adjunct treatment	Ineffective	0.0	0	0	1	31090070
Mastitis	No	Prevention	Effective	100.0	0	33.33 (1/3)	3	32987448
Migraine	No	Prevention	Ineffective	0.0	0	0	1	37042522
Multiple sclerosis	Yes	Adjunct treatment	Ineffective	0.0	0	0	1	32428983
Necrotising enterocolitis	No	Prevention	Ineffective	50.0	0	0	60	37493095
	No	Prevention	Ineffective		0	0	1	37262358
	No	Prevention	Effective		33.33 (2/6)	16.67 (1/6)	6	35230697
	No	Prevention	Effective		33.33 (1/3)	0	3	32232984

Non-alcoholic fatty liver disease	Yes	Adjunct treatment	Ineffective	0.0	17.14 (6/35)	2.86 (1/35)	35	34280304
Organ transplant recipients	No	Adjunct treatment	Ineffective	0.0	0	0	5	36126902
	No	Prevention	Ineffective		0	0	2	24599680
Pancreatitis	Yes	Adjunct treatment	Ineffective	0.0	0	0	5	28431202
	Yes	Adjunct treatment	Ineffective		0	14.29 (1/7)	7	25803695
Post-gastrointestinal surgery	No	Adjunct treatment	Ineffective	0.0	0	0	1	38258877
Preterm labour	No	Prevention	Effective	100.0	n/a	n/a	3	17253567
Preterm mobidity and mortality	No	Prevention	Ineffective	0.0	8.33 (1/12)	8.33 (1/12)	12	30548483
Radiotherapy-associated diarrhea	No	Adjunct treatment	Effective	50.0	12.5 (1/8)	12.5 (1/8)	8	29360138
	No	Prevention, treatment	Ineffective		8.33 (1/12)	33.33 (4/12)	12	30168576
Respiratory infections	No	Prevention	Effective	100.0	0	12.5 (1/8)	8	25344083
	No	Prevention	Effective		11.76 (2/17)	5.88 (1/17)	17	31210358
	No	Prevention	Effective		12.5 (3/24)	4.17 (1/24)	24	36001877
Urinary tract infection	No	Adjunct treatment	Ineffective	0.0	0	0	3	30784039

	No	Prevention	Ineffective		0	22.22 (2/9)	9	26695595
	No	Prevention	Ineffective		0	0	3	28884476
	No	Prevention	**Ineffective, not recommended**		25 (1/4)	0	4	37068952
Vaginosis	No	Treatment	Ineffective	60.0	n/a	n/a	n/a	19821358
	No	Treatment	Ineffective		0	100 (1/1)	1	35005777
	No	Treatment	Effective		10 (1/10)	10 (1/10)	10	29168557
	No	Prevention	Effective		n/a	n/a	1	21833970
	No	Treatment	Effective		0	100 (1/1)	1	19588379

***Conclusions based on low-certainty and low-quality evidence**
